# Using Nuclear Energy for Maritime Decarbonization and Related Environmental Challenges: Existing Regulatory Shortcomings and Improvements

**DOI:** 10.3390/ijerph20042993

**Published:** 2023-02-08

**Authors:** Qiuwen Wang, Hu Zhang, Puxin Zhu

**Affiliations:** School of International Law, East China University of Political Science and Law, Shanghai 201620, China

**Keywords:** nuclear energy, maritime decarbonization, marine environmental challenges, nuclear-powered merchant ships, international regulatory framework

## Abstract

In recent years, the use of nuclear energy as propulsion for merchant ships has been proposed as a means of promoting the transition toward maritime decarbonization and environmentally sustainable shipping. However, there are concerns that nuclear-powered merchant ships could pose risks to the marine environment in the event of accidents, such as collisions, machinery failure or damage, fire, or explosions. The current international regulatory framework for nuclear-powered merchant ships is insufficient to address these risks. This research aims to address this gap by conducting a policy analysis of the existing regulations and a critical examination of their effectiveness in addressing the environmental risks of nuclear-powered merchant ships. Through this analysis, the study identifies the shortcomings and insufficiencies in the current framework and explores potential solutions to improve it, with the goal of enhancing the international community’s ability to mitigate the potential impacts of radioactive marine pollution from nuclear-propelled ships in an era of maritime decarbonization.

## 1. Introduction

Emissions from maritime transport are considered a significant source of marine atmospheric pollution [1]. In the process of promoting the transition toward maritime decarbonization and green shipping in recent years, using nuclear energy as propulsion for merchant ships has been considered a feasible and promising alternative to traditional fossil marine fuels [2,3,4,5,6]. Nuclear energy offers a promising solution for decarbonizing the maritime industry. As a reliable and low-carbon energy source for ships, it has the potential to greatly reduce emissions from shipping. Unlike traditional fossil fuel-powered ships, nuclear-powered ships emit significantly fewer greenhouse gases and air pollutants, positioning them as a cleaner and more sustainable choice for the industry. “Nuclear ship propulsion during operation emits no CO_2_, NO_X_, SO_X_, or particulate emissions” [7]. Using nuclear propulsion could also avoid the occurrence of maritime oil spills, which occur frequently [8]. Moreover, as the scaling up of the production of green ammonia, methanol, and hydrogen has met technical hurdles, the shipping industry has been increasingly interested in nuclear power propulsion. Nuclear power is considered to be particularly suitable to fuel ships that are at sea for long periods of time because it limits the need for refueling while producing zero carbon emissions [9].

Traditionally, nuclear power is mainly used on military ships, such as nuclear-powered submarines and aircraft carriers. Only a few countries, such as Russia, the United States, Germany, and Japan, have used nuclear power for merchant ships. However, in recent years, as maritime decarbonization has become increasingly prominent on countries’ agendas, many shipping companies have begun to explore ways to promote the application of nuclear energy in merchant ship propulsion. For example, the American company TerraPower (Bellevue, WA, USA) has been designing “next-generation” nuclear power plants and exploring their use to propel merchant ships [10]. The British company Core Power (City and County of Denver, CO, USA) was established in 2018 to develop advanced nuclear reactor technologies used for maritime transport [11]. China plans to develop nuclear-powered ice-breaking comprehensive support ships and deploy twenty floating nuclear power plants in the South China Sea [12,13]. Samsung Heavy Industries (Seongnam, Republic of Korea), South Korea’s shipbuilding giant, announced that it will join forces with the Korea Atomic Energy Research Institute (Daejeon, Republic of Korea) to jointly develop molten salt reactors for marine propulsion [14]. Together, Japanese shipbuilding companies and Det Norske Veritas (DNV) have completed the conceptual design of a 20,000-TEU ultra-large container ship equipped with a nuclear fusion reactor [15]. Some countries have begun to develop legislation pertaining to nuclear merchant ships. For example, the United Kingdom enacted Merchant Shipping (Nuclear Ships) Regulations in 2022. Although, for a long time, nuclear power was not regarded as a viable option for merchant ships, nuclear-powered merchant ships have once again captured the attention of major maritime powers due to the necessity of maritime decarbonization.

However, although using nuclear energy to propel merchant ships can generate quality power and almost zero emissions, it may pose other unique risks to the marine environment. Although there are several existing international conventions in place to regulate nuclear-propelled ships, many issues remain about the regulation of potential environmental risks related to such ships. The relevant international regulatory framework has seemingly failed to keep pace with the adoption of nuclear energy for merchant ship propulsion; moreover, these regulations are insufficient to cope with the specific environmental risks induced by nuclear-powered merchant ships.

Against this background, this study aims to address the following three main questions: (1) What are the conventions, regardless of whether they have entered into force, that constitute the existing international regulatory framework for nuclear-powered merchant ships? (2) Is the existing international regulatory framework well equipped to effectively address the specific risks that nuclear-powered merchant ships pose to marine environments? (3) How can we resolve the insufficiencies embedded in the existing international regulatory framework and improve that framework to ensure that the use of nuclear power to propel merchant ships can better anticipate the era of maritime carbon neutrality? This research primarily uses a policy analysis approach to review and examine the conventions, agreements, and protocols in place that constitute the current international regulatory framework for nuclear-powered merchant ships. The study also analyzes the shortcomings and insufficiencies within that framework. Hence, this study explores possible solutions for improving the existing international regulatory framework as this framework addresses the risks that nuclear-powered merchant ships present to the marine environment.

## 2. The Use of Nuclear Propulsion in Merchant Ships

**Figure 1:**
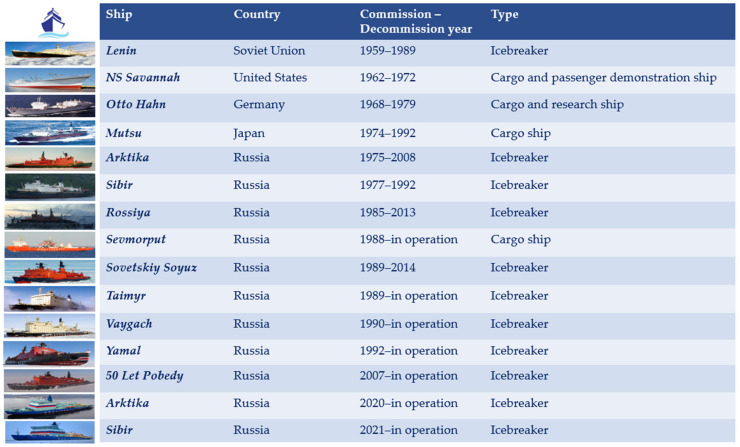
The development of nuclear-propelled merchant ships.

Nuclear power began to be used to propel ships in the 1940s, mainly in military ships. To date, the naval fleets of the US, Russia, China, the UK, France, and India have adopted nuclear propulsion [9]. However, the use of nuclear propulsion in merchant ships used for civilian purposes has developed relatively slowly, and it is mainly used for icebreakers (Figure 1 and Table 1). Compared with conventional icebreakers propelled by fossil fuels, nuclear-propelled icebreakers present many technical and economic advantages, especially when used in polar regions. Such icebreakers have the capacity to “break ice up to three meters thick, navigate without refueling for several months, and thus prolong navigation along the Northern Sea Route for up to ten months of the year” [16]. The Lenin, a Soviet icebreaker commissioned in 1959, was the world’s first nuclear-powered ship to be used for civilian purposes. It was powered by “three OK-150 reactors which were subsequently replaced by two 171 MWt OK-900 reactors” after the reactors were damaged during refueling. The ship was decommissioned in 1989 due to the thinning of its hull [9]. After the Lenin, Russia developed a series of nuclear-powered icebreakers, including Arktika, Sibir, 50 Let Pobedy, Rossiya, Sovetskiy Soyuz, Yamal, Taymyr, and Vaygach, which gave the country an important role in the worldwide history of nuclear-powered icebreaker development [6,9].

In 1962, the US-built NS Savannah (Baltimore, MD, USA), a cargo and passenger demonstration ship powered by a nuclear reactor, was delivered. During its time in operation, the Savannah fulfilled its mission of demonstrating the technical safety and feasibility of nuclear-powered merchant ships. It “travelled 450,000 nautical miles on nuclear power, with one refueling” and “visited ports in 29 countries but was excluded from ports in Australia, New Zealand and Japan” [3]. However, due to the relatively high price of its construction and operation, it was decommissioned after ten years of operation [17]. The Savannah remains a historic landmark of the Atoms for Peace program [18].

In 1968, the German-built Otto Hahn, a nuclear-powered cargo and research ship, was put into operation and sailed 650,000 nautical miles on nuclear power [17]. It had been tested under extremely harsh sea conditions and had successfully achieved its research objective. Although free of technical defects, the Otto Hahn faced restricted routes due to regulatory problems; for example, it was denied access to certain ports on the grounds of nuclear risks. As their operating costs were high, the ship’s nuclear reactors were removed in 1979. The Otto Hahn was then converted into a conventional diesel-powered ship [8].

In the 1970s, Japan’s Mutsu, a nuclear-powered cargo ship, began operating [9]. Japan has long been interested in the application of nuclear energy in merchant ship propulsion since the country lacks domestic energy and has a tight transportation sector. The Mutsu was originally built as a nuclear-powered general cargo ship, and construction started in the late 1960s. However, on its maiden voyage in 1974, “the reactor started to leak fast neutrons, leading to massive protest from local inhabitants, port authorities and fishermen so that the ship was denied return to its homeport, the city of Mutsu. The project failed in respect of technology, public communications and trust” [3,19]. In the face of these technical and political problems, the reactor of the Mutsu was deactivated in 1992, and the ship was converted into an ocean observation ship with diesel engines [3].

In 1988, Russia’s nuclear-powered Sevmorput was delivered. It is a “LASH-carrier (taking lighters to ports with shallow water) and container ship with ice-breaking bow capable of breaking 1.5 metres of ice” [9]. However, after the Chernobyl disaster in 1986, the Sevmorput was denied entry into some ports due to nuclear safety concerns and public protests. The ship is reported to have “operated for over 20 years without major incidents” and is still in service today [3].

In summary, the development of nuclear-powered merchant ships has not been very fast over the past several decades. There are many reasons for this situation. First, the research, development, and construction of nuclear-powered merchant ships are all quite costly, and the daily operation and maintenance costs are also high. Second, serious nuclear accidents such as the Chernobyl disaster have negatively affected the public’s perception of nuclear energy, increasing the public’s fear of such energy sources. Third, regulations relating to nuclear ships are drastically insufficient, exacerbating the public’s lack of confidence in the application of nuclear propulsion in merchant ships. In recent years, with maritime transport’s transition toward maritime decarbonization and green shipping, interest in using nuclear energy as propulsion for merchant ships has been reawakened [4,5]. As mentioned above, many countries such as China, the United States, the United Kingdom, Russia, Japan, South Korea, and Norway have engaged in the research and development of nuclear-powered ships used for civilian purposes. 

## 3. Literature Review, Materials, and Analytical Framework

### 3.1. Literature Review

Transitioning to the use of alternative fuels and energy for ship propulsion has become a true necessity for shipping companies, especially since the International Maritime Organization (IMO) have adopted an initial strategy for reducing greenhouse gas (GHG) emissions from ships [20,21]. The increasing need for cleaner fuels and energy in ship propulsion has stimulated “rising technological advancements in ship nuclear power machinery (SNPM) design” [22]. Many countries have already carried out research and development programs pertaining to nuclear-powered merchant ships; these countries include the United Kingdom, the United States, Russia, China, Japan, South Korea, and Norway [3,17,18,23,24]. Existing research on nuclear energy as propulsion for merchant ships has mainly focused on the following aspects.

First, scholars have noted that nuclear energy can be used as an important alternative energy source for ship propulsion and have examined the advantages and feasibility of using nuclear energy for merchant ship propulsion. Studies have shown that “nuclear fission entails no chemical reactions” and hence can be used as ship propulsion with zero GHG emissions [25,26,27]. Economically, the development of nuclear power systems and their application in maritime transport can be beneficial. Nuclear power allows ships to operate for extended periods without the need for refueling, enhancing their autonomy and insulating them from fuel price fluctuations [8,19]. Research has shown that a key distinction between conventional large container ships and those powered by nuclear energy is in the significant difference in fuel consumption. In comparison, fuel consumption is essentially a non-issue for nuclear-powered ships, which can operate for 15–20 years on a single fueling [28]. Moreover, nuclear-powered ships emit fewer greenhouse gases and air pollutants compared to traditional fossil fuel-powered ships, which can result in lower costs associated with emissions regulations and penalties [29]. However, research has also noted that despite the lower fuel costs, the operational expenses of nuclear ships had proven to be excessively high in prior commercial trials, ultimately resulting in their decommissioning [19]. Technically, experiments have shown that using nuclear propulsion for merchant ships is feasible [3]. The literature has shown that tremendous technological progress has been made in the design and manufacture of marine nuclear reactors, which makes nuclear energy for merchant ship propulsion and power generation feasible and promising [22]. Nuclear energy provides alternative fuel and energy sources for the maritime transport industry, which must urgently reduce its carbon emissions [26]. Researchers have found that nuclear energy can be used as power for merchant ship propulsion, especially for ice-breakers; moreover, nuclear energy sources could replace fossil fuels and become an energy source in the production of other alternative marine fuels such as ammonia, hydrogen, and electricity [30,31,32,33]. Gabbar et al. in 2021 analyzed four different energy systems used for marine propulsion and found that the “nuclear-renewable hybrid energy system” could be “the best energy system for the marine industry to reduce GHG emissions and improve economic performance” [34]. Research on nuclear-powered icebreakers has shown that, compared with fossil fuel-powered icebreakers, icebreakers that use nuclear propulsion, which are widely used in polar navigation, have stronger icebreaking capabilities [3,35].

Second, scholars have also examined the obstacles that emerge in the use of nuclear propulsion on merchant ships. While ammonia, hydrogen, and methanol have been increasingly used for ship propulsion, nuclear power has not thus far been widely considered a viable propulsion option for commercial ships [18,36]. Although nuclear power propulsion has comparative advantages in terms of carbon emissions, existing studies have also pointed to the obvious risks posed by nuclear propulsion. The development of nuclear-powered merchant ships may be restricted by navigation safety risks, the serious consequences of marine accidents, and social-political risks such as public opposition [8,35,37,38,39]. Adumene et al. (2022) noted that “the complex design feature of the nuclear plant and the ship architecture and operational dynamic” may make the monitoring of nuclear propulsion complicated. Hirdaris et al. (2014) identified realistic risks in the application of modern small modular reactor (SMR) technologies in the shipping sector [26]. Fu et al. (2022) employed a probabilistic approach to examine the safety risks of nuclear-powered icebreakers and found that when a collision, machinery failure or damage, fire, or explosion occurs, nuclear-powered icebreakers may experience nuclear leakage, causing great damage to personnel, property, and the marine environment. Scholars have also noted that the wide use of nuclear energy may meet sociopolitical obstacles due to “public opposition and anti-nuclear politics” [23]. In particular, the Fukushima Daiichi accident heightened public safety concerns about the utilization of nuclear energy; this opposition may negatively affect the large-scale application of nuclear energy in maritime transport [16,21,40,41,42,43]. These considerations could restrict the access of nuclear-powered merchant ships to ports [8,34].

Third, existing research has examined possible ways to promote the use of nuclear energy for marine propulsion in the context of maritime decarbonization. Scholars have emphasized improving ships’ structural design to provide a safer operating environment for nuclear reactors and equipping ships with better collision protection devices to “absorb and distribute the energy of impact” [25]. Some scholars have proposed technical innovations such as an optimal design of “supercritical water-cooled reactors” to be used on ships [17]. Researchers have also proposed several targeted risk control options to mitigate the risk of maritime accidents involving nuclear-powered icebreakers; these options include enhancing ship management, reducing human error-induced maritime accidents, strengthening maintenance operations on board, using effective safety systems and devices, and establishing sound contingency plans [35]. Moreover, some scholars have highlighted the importance of effective safety regulations at both national and international levels and the need to bolster a “nuclear safety culture” globally, thereby making the public and policy-makers “better appreciate and respond to the perception of safety risks” [23]. Existing research has also emphasized the necessity of reaching an agreement concerning the rights of flag and port states to ensure that nuclear-powered merchant ships can enter their ports [3].

The research above has shown that the application of nuclear propulsion to nuclear-powered merchant ships is feasible and has pointed to the many technical, economic, and sociopolitical obstacles a widespread application would face. Hence, multidisciplinary studies have provided useful insights for the development of nuclear-powered merchant ships from different perspectives. Among the solutions proposed, improvements in the regulation of nuclear-powered merchant ships are considered to be of great importance. However, concerns about the marine environment constitute a major factor in the dilemma faced by nuclear-powered merchant ships. There is a lack of comprehensive research focusing on the regulatory framework governing the environmental risks generated by nuclear-powered merchant ships and on the existing international regulatory framework, which could be improved to mitigate or prevent these marine environmental risks.

### 3.2. Methods, Materials, and Analytical Framework

Notably, “International law and institutions serve as the main framework for international cooperation and collaboration between members of the international community in their efforts to protect the local, regional and global marine environment” [44]. Therefore, in this research, international law is used as a lens to examine the regulation of radioactive marine pollution caused by nuclear-powered merchant ships. The study is based on a policy analysis of the international legal instruments in place involving the regulation of nuclear-powered merchant ships and their related marine environment risks. The study aims to unveil the shortcomings and insufficiencies embedded in the existing regulatory framework and explore potential and feasible solutions for the further improvement of the framework. The materials used in the study mainly consist of international conventions, resolutions, agreements, and other relevant instruments that may cover the regulation of nuclear-powered merchant ships and issues related to the marine environment. These international legal instruments are collected from the official websites of the United Nations Treaty Collection, International Atomic Energy Agency (IAEA), and IMO.

**Figure 2:**
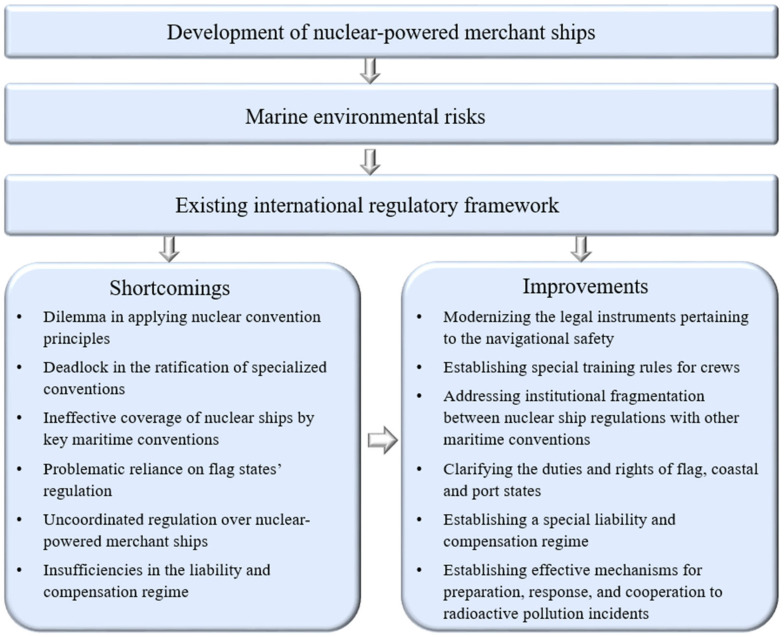
Analytical framework.

In addition to Section 1 and Section 3, the remainder of the research is structured as follows (Figure 2). Section 2 pertains to the application of nuclear power in merchant marine propulsion and the development of nuclear-powered merchant ships in practice. Section 4 covers the marine environmental risks that may arise because of the operation of these ships. Section 5 offers an analysis of the existing international legal instruments that regulate nuclear-powered merchant ships and their marine environmental risks. Section 6 presents how the critical analysis was conducted to understand the insufficiencies embedded in the existing international regulatory framework. Section 7 introduces some potential and feasible solutions for improving the international regulatory regime.

## 4. Nuclear Propulsion Related Marine Environmental Challenges

Using nuclear power to propel ships offers “both benefits for regional economic and social development and risks of nuclear and radiological accidents and concerns about radioactive wastes” [16]. Although the prospect of rapid achievement of maritime decarbonization may be very attractive, nuclear reactors on board ships may also pose potential risks to the marine environment [23,45,46].

First, the daily operation of nuclear-powered merchant ships not only brings environmental concerns of perimeter contamination and thermal pollution but is also not completely free from low-level radiation release. A ship equipped with a nuclear reactor is equivalent to a mobile nuclear installation and carries risks of low-level radiation release, which may cause persistent low-dose radioactive contamination of the marine environment. Fish and marine mammal populations might experience increasing mortality rates, as long half-life isotopes accumulate in the food chain [47]. At the same time, nuclear-contaminated seawater may further contaminate coastal ecosystems, such as mangroves, because of tidal action. Moreover, nuclear reactors on board ships can produce large amounts of heat energy. Thermal pollution arising from nuclear ships may affect marine flora, fauna, and ecosystems in nearby waters as water temperature changes can fatally damage marine life, change the chemical composition of seawater, and cause ecological imbalance [48,49].

Second, nuclear-powered merchant ships that experience maritime accidents can lead to nuclear disasters at sea. In certain circumstances such as ship collision, severe machine damage, fire, or explosions, nuclear-powered merchant ships can experience nuclear leakage, causing severe harm to the marine environment, marine ecosystem, and human health [37]. During its maiden voyage, the Mutsu, a Japanese nuclear-powered merchant ship had experienced a nuclear leakage problem, making local inhabitants, port authorities, and fishermen strongly oppose its continued operation [17]. After emergent nuclear incidents occur at sea, the radionuclides may be dispersed near the sea surface: “The airborne nuclides will diffuse to other area with the atmospheric motion, and cause the environmental pollution and hazardous results to human health” [39]. Although the likelihood of leakage or spillage from an overheated reactor is relatively small, negligence in or the mismanagement of the reactor or the failure of the ship’s nonnuclear components could lead to a rupture of the reactor compartment [50]. Research shows that human errors are responsible for most shipping accidents [51]. The working conditions on board ships are more stressful than those in land-based nuclear installations, and consequently operators of nuclear-powered ships are more prone to make errors that lead to nuclear accidents [52]. Sometimes, nuclear leakage from reactors on board ships result in the emergency dumping of nuclear fuels [16].

Third, radioactive waste from nuclear-powered merchant ships may also be a problem. Although nuclear-powered merchant ships can achieve zero GHG emissions, in the course of their operation, they also generate nuclear waste that may cause radioactive marine pollution. The use of nuclear propulsion poses risks regarding the “safe storage for spent nuclear fuel and decommissioned on-board power plants” [7]. In its first year of operation, the NS Savannah released 115,000 gallons of low-level waste into the ocean [53]. “Radioactive wastes are not biodegradable, nor is there any possibility of removing them from the sea once they have entered it. These substances vary in their effect, but in general, they are absorbed by marine organisms, often becoming concentrated as they move up the food chain, and affecting the growth, reproduction and mortality of marine life” [47]. Research has shown that most of the pollution damage nuclear power does to the environment arises from the process of refueling and the disposal of radioactive wastes [54]. Nevertheless, the potential to reduce waste generation and storage through advancements in nuclear technology remains a promising aspect. Generation IV technology, in particular, has been designed with this objective, incorporating features such as increased fuel utilization and passive safety mechanisms to minimize waste [55,56,57,58]. For instance, sodium-cooled fast reactors can recycle used fuel, thus reducing waste and bringing benefits of integral recycling in nuclear waste management in a closed fuel cycle [59]. Generation IV and other advanced nuclear technologies present a promising outlook for addressing waste management challenges in the future.

These special marine environmental risks require a comprehensive and sound international regulatory framework for the development and operation of nuclear-powered merchant ships, thereby enhancing the ability of the international community to effectively supervise the operation of nuclear-powered merchant ships; ensure the safe disposal of nuclear waste; and prevent, prepare for, and respond to these risks.

## 5. Existing International Regulatory Framework for Nuclear Propulsion Related Marine Environmental Risks

The current international regulatory framework that applies to nuclear-powered merchant ships and their marine environmental risks involves a series of international legal instruments, including not only the conventions, resolutions, and protocols adopted under the auspices of the United Nations, IMO, and IAEA concerning maritime transport safety, nuclear safety, and radioactive marine pollution control (Table 2) but also bilateral agreements negotiated between states, particularly for the passage and port access of nuclear-powered merchant ships.

With regard to the regulation of nuclear-powered merchant ships’ marine pollution risks, conventions for marine pollution control and marine environment protection can be applied generally. The 1982 United Nations Convention on the Law of the Sea (UNCLOS) plays a fundamental role in preserving and protecting the marine environment and preventing its pollution from radioactive sources. In terms of the regulation of “foreign nuclear-powered ships” and “foreign ships carrying nuclear materials”, UNCLOS allows coastal states to restrict these ships’ innocent passages in the territorial sea. These ships are required to confine passage to designated sea lanes, carry necessary certificates, and “observe specific precautionary measures in accordance with international agreements” [60]. UNCLOS also establishes a general framework for the regulation of vessel-source pollution that is applicable to the marine pollution induced by nuclear-powered merchant ships. States have the general obligation to “protect and preserve the marine environment” and must adopt necessary measures to “prevent, reduce and control pollution of the marine environment from any source” [60]. The convention also provides for the jurisdictional rights and obligations of flag, coastal, and port states pertaining to the regulation of marine environmental pollution, including the right to formulate international rules and standards, laws, and regulations and to adopt necessary measures to ensure their effective implementation [60]. When marine environmental damage occurs, states are responsible for ensuring prompt and appropriate compensation and remedies [60]. Moreover, the 1973 International Convention for the Prevention of Pollution from Ships (MARPOL) has provisions for the prevention and control of marine pollution by harmful substances discharged by ships and for the minimization of the pollution produced by shipping accidents [61]. These provisions help protect the marine environment from pollution by radioactive pollutants from various sources on ships. In addition, if nuclear leakage from reactors on board ships results in an emergency dumping of nuclear materials [16], the 1972 Convention on the Prevention of Marine Pollution by Dumping of Wastes and Other Matter (London Convention) may also apply. The convention requires state parties to take practicable measures to protect the marine environment from damage caused by radioactive pollutants and establish a licensing system for radioactive waste disposal [62].

There are also some international conventions and resolutions that specifically set rules for the navigation safety and pollution responsibilities of nuclear-powered merchant ships, including the 1962 Convention on the Liability of Operators of Nuclear Ships (Brussels Nuclear Ship Convention), the 1974 International Convention for the Safety of Life at Sea (SOLAS Convention), and the 1981 Code of Safety for Nuclear Merchant Ships. The Brussels Nuclear Ship Convention aims to address liability issues and establish uniform rules for all nuclear ship operators. Different from conventions about nuclear safety and liability, the Brussels Nuclear Ship Convention is a specialized international convention specifically focused on setting rules and standards for nuclear-powered ships. It imposes exclusive and strict liability on the operator of these ships, provides limitations on the liability of the operator, and allows victims to choose “either the court of the licensing state or the court of the contracting party on whose territory the nuclear damage was sustained” as the forum for dispute resolution [63,64]. Therefore, the convention provides clearer guidelines for nuclear-powered merchant ships and avoids disputes about the applicability issue that bothers traditional nuclear liability conventions’ application in the regulation of these ships [65,66]. However, the convention failed to enter into force due to insufficient ratification numbers. The SOLAS Convention has a special chapter on nuclear ships, specifically stipulating the navigation safety requirements for nuclear-powered ships [67]. The IMO also adopted the Code of Safety for Nuclear Merchant Ships, which aims at “providing an internationally accepted safety standard for the design, construction, operation, maintenance, inspection, salvage and disposal of nuclear merchant ships” [68]. The code only applies to nuclear ships propelled with a “pressurized water reactor”, which is the most common type of reactor for marine prolusion purposes [36,68].

Furthermore, nuclear-powered merchant ships might also be subject to the regulation of international nuclear safety and nuclear liability conventions adopted under IAEA auspices, including the 1960 Convention on Third Party Liability in the Field of Nuclear Energy (Paris Convention), the 1963 Vienna Convention on Civil Liability for Nuclear Damage (Vienna Convention), the 1963 Convention Supplementary to the Paris Convention (Brussels Supplementary Convention), the 1994 Convention on Nuclear Safety, and the 1997 Convention on Supplementary Compensation for Nuclear Damage (CSC). If involved in the disposal of spent fuel and radioactive waste, nuclear material management, or the notification and handling of nuclear incidents, nuclear-powered merchant ships might also be subject to the 1979 Convention on the Physical Protection of Nuclear Material, the 1986 Convention on Early Notification of a Nuclear Accident, the 1986 Convention on Assistance in the Case of a Nuclear Accident or Radiological Emergency, and the 1997 Joint Convention on the Safety of Spent Fuel Management and on the Safety of Radioactive Waste Management. These conventions have established several important principles in dealing with nuclear safety and liability issues, such as channeling liability exclusively to the operator, imposing supplementary compensation liability on the installation state, requiring the providence of financial warranties and mandatory insurance, setting limitations for liability, and granting exclusive judicial jurisdiction to the state in which a nuclear accident occurs. However, applying these principles to nuclear-powered merchant ships may not only encounter several practical difficulties but also face application restrictions arising from the conventions’ application scope [64,69,70]. Some conventions “assign the ultimate responsibility for nuclear safety to individual countries”, indicating a lack of “effective enforcement measures to ensure compliance such as imposing sanctions in the case of non-compliance” [23,71].

Additionally, states have negotiated bilateral agreements particularly for dealing with port access and territorial water passage issues for nuclear-powered merchant ships. For example, the 1964 USA-UK Agreement relating to the Use of United Kingdom Ports and Territorial Waters by the N.S. Savannah, the 1968 Agreement between the Federal Republic of Germany and the Kingdom of Netherlands on the Use of Dutch Waters and Harbors by the NS Otto Hahn, and the 1970 Treaty between the Federal Republic of Germany and Liberia on the Use of Liberian Waters and Ports by the NS Otto Hahn are such bilateral agreements. These bilateral agreements negotiated between states address issues such as the use of coastal state waters, port access, and liability and compensation for nuclear incidents [47]. However, these agreements are ship-specific and state-specific and do not have binding effects for other nuclear-powered merchant ships and third-party countries.

## 6. Shortcomings in the Existing International Regulatory Framework

**Figure 3:**
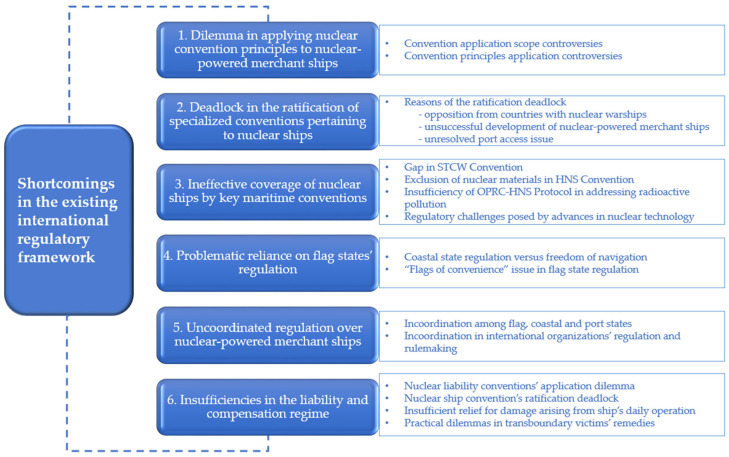
Shortcomings in the existing international regulatory framework.

Governments have again become interested in developing nuclear-powered merchant ships to fulfill maritime decarbonization requirements; however, the relevant international regulatory framework has seemingly failed to keep pace with the use of nuclear propulsion in maritime shipping. The many international conventions and protocols in place constitute a rather complex institutional framework for the regulation of nuclear-powered merchant ships. The institutional complexity of the framework and several shortcomings and insufficiencies within it may cause difficulties in effectively regulating nuclear-powered merchant ships and managing the environmental risks they pose to the sea (Figure 3).

### 6.1. Dilemma in Applying Nuclear Convention Principles to Nuclear-Powered Merchant Ships

A series of international conventions and protocols concerning nuclear safety and nuclear liabilities have been adopted under the auspices of the IAEA. These international legal instruments have established a number of principles in the fields of nuclear safety regulation and nuclear liability and compensation. Although some scholars have disagreed with the “broader interpretation” of existing conventions’ application scope to include transportable nuclear installations [66], other scholars have opined that nuclear liability conventions provide room for such interpretation and can be applied to floating nuclear power platforms and nuclear ships [69]. However, the application or transplantation of these principles primarily designed for land-based nuclear installations to nuclear-powered merchant ships may encounter several problems.

Under the existing nuclear liability regime, strict and exclusive liability has been imposed on the operators of nuclear installations [72,73]. However, the application of the operators’ exclusive liability principle in nuclear-powered merchant ships may generate several difficulties. First, the assumption of the operators’ liability does not fit into the operation and management of nuclear-powered merchant ships because ships are not managed only by their operators. Indeed, both owners and managers may be responsible for the management of ships. Thus, this principle may exonerate shipowners from liabilities [21]. Second, strict and exclusive liability channeling may also face practical problems in its application to nuclear-powered merchant ships. Considering the high level of compensation for nuclear damage, if the ship owner/operator were required to assume no-fault liability, it might seriously hinder the development of nuclear-powered merchant ships because almost no owner/operator would be willing to bear the cost of nuclear accidents simply to reduce carbon emissions. As noted by Vlajić, applying the strict liability principle to all damages may not be reasonable because compared with immobile nuclear facilities, nuclear ships are exposed to more external instability factors “such as temperature oscillation, wind and water resistance, collision, corrosion” [74]. Third, exclusive liability has also been criticized for freeing “suppliers and other service providers from liability and compensation” [75]. Fourth, natural disasters are often evoked as reasons for liability exemption under existing nuclear liability conventions [72,73,76]; however, this rationale may lead to liability exemption in many cases because the probability of nuclear ships encountering natural disasters, particularly while sailing at sea, is much greater than that of land-based nuclear installations.

Furthermore, the assumption of supplementary liability by the states in which installations have taken place and their exclusive jurisdictions over nuclear damage claims may also create problems in the application of the law to nuclear-powered merchant ships. The existing nuclear liability regime channels supplementary liabilities to the states that have made the installations. However, channeling state liabilities to the nuclear-powered merchant ships’ flag states could be quite problematic if only a “flag of convenience” is considered. The issue of a “flag of convenience” may undermine the state’s supplementary financial security [21]. Additionally, applying the principle of exclusive judicial jurisdiction to the state within which a nuclear incident occurs could create problems in the case of nuclear-powered merchant ships. If a nuclear-powered merchant ship leaks into a country’s coastal waters, requiring victims to claim compensation in the court of the ship’s flag state is not only unreasonable but also unjust.

Additionally, the remedies available under existing conventions are mostly premised on the damage caused by nuclear incidents. However, the marine environment may become polluted through the daily operations of nuclear-powered merchant ships and remedies and compensation for such pollution damage have been largely overlooked.

### 6.2. Deadlock in the Ratification of Specialized Conventions Pertaining to Nuclear Ships

Although the Brussels Nuclear Ship Convention specifically focused on addressing liability issues for nuclear-powered ships, its ratification has turned into a deadlock. The convention failed to enter into force because of insufficient ratification numbers. The ratification deadlock can be attributed to several reasons [64,77]. First, the endeavor to include nuclear-powered naval ships in the convention aroused strong opposition from countries with nuclear-powered warships already in operation and development. These countries feared that including warships in the application scope of the convention “might presage an attempt to impose other types of regulations on these ships, e.g., international inspections or licensing requirements” [64] and insisted that rules for military ships have no place in a civil liability convention [77,78,79,80]. Second, the development of nuclear-powered merchant ships and their use in maritime transport was still limited at the time. Third, the convention did not address the issue of the nuclear ships’ entrance into ports. The convention stipulates that its clauses would not affect coastal and port states’ rights to deny nuclear-powered ships access to their waters and harbors [63]. This stipulation meant that the parties would have to negotiate special bilateral agreements to obtain the right to enter ports for their nuclear ships [64]. These factors all negatively affected the contracting parties’ active participation in the convention ratification process.

The ratification deadlock has undermined the efforts of the international community to create a multilateral framework regulating nuclear ships. The factors that hindered the convention’s ratification may also become obstacles to the formulation of multilateral rules for nuclear-powered merchant ships with wide international acceptance and active state participation today and in the future.

### 6.3. Ineffective Coverage of Nuclear Ships by Key Maritime Conventions

The operation of nuclear-powered merchant ships requires special qualifications and training of seafarers, prompt and effective response to radioactive accidents on board ships, and incorporating new technological developments related to nuclear reactors into relevant regulatory frameworks. However, although some important maritime conventions have responded to these issues, there are still certain gaps and deficiencies in whether they can effectively cover nuclear-powered merchant ships.

Seafarer qualifications and training are essential not only to ensure the safety of nuclear-powered merchant ships and prevent nuclear pollution accidents but also to protect the safety of the seafarers. Nuclear-powered merchant ships pose unique challenges due to potential collisions and close proximity of operating personnel. It has been recommended by experts to follow the International Commission on Radiological Protection’s guidelines for maximum permissible radiation doses, treating all crew as “radiation workers” and setting maximum yearly radiation dose for crew members [53]. Indeed, the 1978 International Convention on Standards of Training, Certification and Watchkeeping for Seafarers (STCW Convention) establishes a series of mandatory training, certification, and watchkeeping requirements for seafarers, which must be fulfilled by all participating countries. However, the special qualifications and training of seafarers on board a nuclear ship are not currently addressed in the STCW Convention [81].

Effective mechanisms for the preparation, response, and cooperation among states in dealing with maritime accidents involving nuclear-powered merchant ships are also vital. The 1996 International Convention on Liability and Compensation for Damage in Connection with the Carriage of Hazardous and Noxious Substances by Sea (HNS Convention) and the 2000 Protocol on Preparedness, Response and Co-operation to Pollution Incidents by Hazardous and Noxious Substances (OPRC-HNS Protocol) seek to establish a global framework for issues of liability, compensation, and international cooperation in addressing major incidents or threats of marine pollution caused by hazardous and noxious substances. However, loss or damage caused by certain categories of radioactive materials is excluded by the HNS Convention and the convention has not yet come into force [82]. Some response measures and the organizational structure for pollution control in the OPRC-HNS Protocol follow the principles of the OPRC Convention, which is an oil pollution convention. It has been raised as a concern that conventional oil spill resources may not be adequate for responding to spills of radioactive materials [83].

The advancement of nuclear energy technology presents opportunities for its application in other fuel type ships and the integration of alternative fuels into nuclear ships. For example, nuclear energy could become an energy source in the production of other gas fuels such as ammonia and hydrogen [30,31,32,33]. It is not impossible that dual-fuel ships utilizing both nuclear and ammonia/hydrogen energy will emerge in the future. All these nuclear technological progresses imply that the development of nuclear technologies may result in nuclear-powered ships being bound by the regulations of gas fuel conventions such as the 2015 International Code of Safety for Ships Using Gases or Other Low-Flashpoint Fuels (IGF Code). However, the fragmented regulations governing nuclear ships and other alternative fuel ships pose a challenge for international institutions to effectively manage the advancements in these technologies.

### 6.4. Problematic Reliance on Flag States’ Regulation

Nuclear-powered merchant ships’ navigation safety and pollution control are governed by a series of existing conventions. However, there are still many deficiencies in the existing international regulatory framework. Under the existing international regulatory framework, coastal states play only a limited role in the regulation of nuclear-powered merchant ships. The regulation over these ships mainly relies on the flag state. Considering the safety and environmental threats that nuclear-powered ships may bring to coastal states, the UNCLOS specifically stipulates regulatory measures that coastal states can take when such ships pass through their territorial waters; these measures include designating “sea lanes and traffic separation schemes” for foreign nuclear-powered ships, requiring these ships to present certain documents, and requiring them to “observe special precautionary measures established by international agreements” [60]. However, a coastal state’s ability to control issues of pollution prevention when faced with a nuclear-powered merchant ship is largely limited to that state’s territorial waters. A nuclear-powered merchant ship navigating inside exclusive economic zones is able to enjoy freedom of navigation, which leaves regulatory power over these ships to the flag states. Although “coastal states have jurisdiction with regard to the protection and preservation of marine environment” in the exclusive economic zones, whether such jurisdiction indicates that coastal states can take regulatory measures against nuclear-powered merchant ships in the exclusive economic zones on the grounds of marine environmental protection remains uncertain and may lead to disputes among states in practice [84].

SOLAS has introduced a special chapter that ensures the navigation safety of nuclear ships, requiring that flag states “take measures to ensure that there are no unreasonable radiation or other nuclear hazards, at sea or in port, to the crew, passengers or public, or to the waterways or food or water resources” [67]. However, because of the “flag of convenience” issue, flag states’ control is deficient and regulations are ineffective. Furthermore, the existing legal framework places the responsibility of oversight on the high seas in the hands of flag states. Nevertheless, “experience shows flag states often fail to provide adequate oversight with so-called ‘flags of convenience’ offering low-cost registration, loose environmental and operational requirements, and weak enforcement” [85]. There is currently no international agency or organization that can effectively monitor the radioactive waste disposal on the high seas [86].

### 6.5. Uncoordinated Regulation over Nuclear-Powered Merchant Ships

Existing regulatory frameworks have failed to establish effective coordination in the regulation of nuclear-powered merchant ships. Nuclear-powered merchant ship navigation has been subject to the regulations of flag, coastal, and port states. In the absence of effective coordination, the navigation management and pollution control of nuclear-powered merchant ships face fragmentation problems, which may hinder the development and large-scale use of such ships in the future. Unlike nuclear-powered warships, nuclear-powered merchant ships’ purpose is transporting goods and people. Therefore, entrance into ports has become an inevitable problem in navigation [87]. This means that nuclear-powered merchant ships must obtain regulatory certifications from the flag states and port states they visit to secure their voyage and port entry [18,88]. In the absence of regulatory coordination among flag states, port states, and coastal states, nuclear-powered merchant ships may face great obstacles. In particular, considering that nuclear-powered merchant ships would pose a major threat to the marine environment if a marine accident were to occur, making port and coastal states accept these ships is difficult. The 1962 Brussels Nuclear Ship Convention does not address this issue and leaves the right to deny access to their ports [63]. This difficulty has contributed to the convention’s ratification deadlock. To solve such incoordination and facilitate the navigation of nuclear-powered merchant ships, some scholars have highlighted that “before possible further experimenting with nuclear merchant ships, it is necessary to reach agreement upon the rights of the flag and port state” [3].

In addition to this lack of regulatory coordination among flag, coastal, and port states, incoordination also exists in international organizations’ regulation and rulemaking. The IMO’s regulation and rulemaking have focused mainly on nuclear ships’ navigational safety, while the IAEA’s focus has mainly been on nuclear safety and post-accident liability and compensation issues. Research has shown that the IMO often “lacks effective implementation of existing international conventions, which results in a situation of growing regulatory fragmentation, —e.g., regional regulations—and uncertainty” and the Code of Safety for Nuclear Merchant Ships has not been implemented [3,21,89,90]. IAEA dominance in nuclear accident investigations could create further fragmentation. For example, after a nuclear ship leakage accident, if the investigation were mainly supervised by the IAEA, as was the case in Japan’s nuclear wastewater disposal after the Fukushima nuclear accident, it may result in the inadequate participation of other international organizations, such as the IMO and the Food and Agriculture Organization in the protection and preservation of the marine environment [91]. Fragmentation may make it difficult to effectively address nuclear-powered merchant ships’ marine environmental risks. This situation therefore calls for “further coordination and integration of the rules between the IMO and IAEA to solve the potential regulatory dilemma for nuclear energy ships” [21].

### 6.6. Insufficiencies in the Liability and Compensation Regime

When nuclear-powered merchant ships cause radioactive marine pollution, effective accountability of responsibility and victims’ “access to justice based on prompt and adequate compensation” are foundational to the relief process [76]. However, in regard to the radioactive marine pollution caused by daily operations or a sudden accident involving nuclear-powered merchant ships, victims might face practical problems in seeking relief under existing international regulatory frameworks.

First, although liability regimes have been established through a number of nuclear liability conventions, their scopes of application are not all inclusive. Whether these conventions can be applied to nuclear-powered merchant ships is controversial. If interpreted strictly, many important nuclear liability conventions may solely apply to “land-based nuclear installations” and therefore exclude nuclear-powered ships [66]. Furthermore, the 1971 Convention Relating to Civil Liability in the Field of Maritime Carriage of Nuclear Material “exonerates others who may be responsible for damage caused by a nuclear incident if the operator of a nuclear installation is held liable for such damage under either the Paris or the Vienna Convention or under national law” [92]. Additionally, although the Brussels Nuclear Ship Convention attempted to address nuclear ship related liability issues, the ratification of the convention has fallen into a deadlock [64].

Second, the remedies available under existing conventions are mostly premised on the damage caused by nuclear incidents. However, the marine environment can also be polluted through the daily operation of nuclear-powered merchant ships. Compensation for the adverse impact of these daily operations on marine flora, fauna, and ecosystems is not effectively covered by the liability and compensation schemes included in existing nuclear liability conventions. The existing international regulatory framework also lacks an effective mechanism for monitoring the radiation of nuclear merchant ships in their daily operations.

Third, under existing nuclear liability conventions, transboundary victims’ remedies also face practical dilemmas. Under the existing liability regime, compensation for transboundary damage is often premised on the “knowledge and foreseeability of the risk” [93,94,95]. This implies that marine pollution caused by unforeseen reasons such as maritime disasters involving nuclear ships may not necessarily be fully compensated. Another element that may limit compensation is the “significant damage” threshold. The International Law Commission’s Draft Principles on the Allocation of Loss in the Case of Transboundary Harm Arising out of Hazardous Activities and several landmark cases require that the “damage eligible for transboundary compensation” must be “significant damage” to the environment [96,97,98]. Simply showing the risk of potential harm may not be sufficient to entitle the affected parties to legal relief [99,100]. Consequently, these legal thresholds might present hurdles for the victims’ compensation claims in cases in which marine environment pollution was induced by nuclear-powered merchant ships [101].

## 7. Potential Ways to Improve the Existing Regulatory Framework

The development of nuclear propulsion for merchant ships provides an alternative choice of maritime fuel in an era in which maritime decarbonization has gained momentum. However, this use of nuclear power may also bring many challenges to the international community in terms of nuclear safety protection and radioactive marine pollution control. The insufficiencies of the existing international regulatory framework may make it difficult to address these challenges. As mentioned above, the current international regulatory framework for nuclear-powered merchant ships has flaws in terms of radioactive pollution prevention and control. Coastal states’ ability to regulate under the UNCLOS has been largely confined to their territorial waters. SOLAS’s reliance on flag state control may generate practical enforcement difficulties due to the issue of “flags of convenience”. Existing conventions on nuclear safety and liability are not inclusive in their scope of application and may apply only to land-based nuclear installations [66]. The ratification of the Brussels Nuclear Ship Convention is deadlocked. Several important maritime conventions that are valid and popular on the decarbonization method of maritime transport may not effectively cover nuclear-powered merchant ships. The shortcomings and insufficiencies within the current international regulatory framework have impeded effective pollution prevention and control of nuclear-powered merchant ships.

Therefore, to effectively regulate nuclear-powered merchant ships and their marine environmental risks, it is necessary to establish a relatively comprehensive multilateral framework, thereby providing institutional guidance for the navigational safety of these ships; preventing and controlling their pollution to the marine environment; and ensuring prompt, effective, and adequate compensation after nuclear accidents. At present, the IMO is dedicated to promoting the development of international regulations on the use of alternative marine fuels and energy and has established rules for a number of gas fuels, such as liquefied natural gas, methyl/ethyl alcohol fuel, hydrogen, ammonia, etc. [102]. Regulations applying to nuclear-powered merchant ships, the propulsion of which constitutes an alternative fuel in light of maritime decarbonization, can also be formulated under the auspices of the IMO together with IAEA, which would solve the problem of fragmentation and inconsistency that has dominated rulemaking and regulations.

The formulation of a comprehensive multilateral international framework for the regulation of nuclear-powered merchant ships and their environmental risks could be premised on the reform, modernization, and synthesization of existing international legal instruments. This would involve, first, modernizing the legal instruments pertaining to the navigational safety of nuclear-powered merchant ships to mitigate their pollution risks caused by maritime accidents. Although the nuclear ship chapter in the SOLAS Convention and Code of Safety for Nuclear Merchant Ships can be used as a set of requirements for the safe navigation of nuclear-powered merchant ships, further improvements are needed. It has been widely agreed that the nuclear ships chapter in the SOLAS Convention and the Code of Safety for Nuclear Merchant Ships need to be substantially modernized to effectively keep pace with the application of nuclear technology in marine propulsion in the era of maritime decarbonization. Deficiencies, such as the reliance on regulations by flag states, the lack of “standards for the design, operation and development of nuclear merchant ships”, and the restricted application scope which includes only “pressurized water reactors” and excludes other types of nuclear reactors, need to be addressed [3,6,103]. Second, considering that human errors are responsible for most shipping accidents [51], establishing special training rules for crews on board nuclear-powered merchant ships is also important [74]. The STCW Convention could potentially include special requirements for nuclear ship crew qualifications and training as a means of improving the current international framework. Third, to address the lack of coordination in regulations among flag, coastal, and port states, facilitate the navigation of nuclear-powered merchant ships, and ensure their port access and access to maritime search and rescue in emergency cases, it is necessary to clarify the duties and rights of flag, coastal, and port states [3]. Fourth, a special liability and compensation regime for nuclear-powered merchant ships should be established. The Brussels Nuclear Ship Convention could be used as an initial draft. Nevertheless, stumbling blocks that had negatively affected convention ratification, such as the ambition to bring nuclear warships under the scope of application, must be abandoned to enhance contracting parties’ willingness to ratify the convention. Moreover, adopting mandatory insurance requirements and compensation fund mechanisms is also important, as it could “ensure prompt, effective and adequate compensation for victims of nuclear damage” [91]. Fifth, the nuclear technology developments may present opportunities for nuclear energy’s application in other fuel type ships, the integration of alternative fuels into nuclear ships, or even the development of dual-fuel or multi-fuel ships and thereby involving the simultaneous application of different fuel-type-specific conventions. However, the current fragmented regulations will not be effective in coping with the advancements in technology in the future. Hence, as technology continues to advance, addressing institutional fragmentation through harmonizing existing regulations or introducing new legislation may become an urgent necessity. Sixth, it may also be useful to establish effective mechanisms for the preparation, response, and cooperation of contracting parties in addressing radioactive marine pollution accidents involving nuclear ships, perhaps under the OPRC-HNS Protocol framework, thereby enhancing the international community’s capabilities in dealing with pollution incidents induced by nuclear-powered merchant ships. “Emergency prevention, preparedness, and response” are essential to the global governance of nuclear accidents. Therefore, establishing relevant arrangements to institutionalize and streamline prevention, preparedness, and response procedures for accidents involving nuclear-powered merchant ships and to promote cooperation among contracting states is important. Such procedures could assist with accident notification, maritime search and rescue, the monitoring of radionuclides diffusion in the ocean, and the cleaning up of radioactive contamination [16].

## 8. Conclusions

In promoting the transition toward maritime decarbonization and green shipping, the use of nuclear energy as propulsion for merchant ships has been considered a feasible and promising alternative to traditional fossil marine fuels in recent years. The use of nuclear power as propulsion in maritime transport can help reduce carbon emissions, but it may induce other risks for the marine environment. Nuclear-powered merchant ships’ collisions, severe machinery damage, fires, explosions, or nuclear leakage may cause serious harm to the marine environment. Current research on nuclear propulsion for merchant ships has shed light on the technical, economic, and sociopolitical challenges to widespread adoption. However, despite the valuable multidisciplinary insights, there remains a deficit in thorough and in-depth research from an international law perspective. This highlights the need for further examination of the international regulatory framework governing the environmental risks posed by nuclear-powered merchant ships. Although there are many international conventions, protocols, and agreements in place that regulate the use of nuclear propulsion in merchant ships, this study has shown through a critical analysis that the current international regulatory framework is riddled with insufficiencies that prevent the environmental challenges posed by nuclear-powered merchant ships from being effectively regulated. These insufficiencies and shortcomings include issues with the application of nuclear convention principles, the ratification deadlock of specialized conventions for nuclear ships, a problematic reliance on flag states’ regulation and regulation incoordination, and inadequate liability and compensation mechanisms for nuclear-powered merchant ships’ environmental damage indemnity. This study presents options for improving the existing international regulatory framework. It is hoped that this study critically examining the existing international regulatory framework from an international law perspective could provide some insights into future research on the regulation of nuclear-powered merchant ships, the use of nuclear propulsion for maritime decarbonization, and radioactive marine pollution control, as well as some reflections on a series of issues concerning the role that law and policy plays in shaping the development of nuclear-powered merchant ships and advanced nuclear technologies, such as: does the current legal and policy framework promote or hinder the development of nuclear-powered merchant ships? How can the use of nuclear propulsion contribute to the decarbonization of the maritime industry, particularly within the framework of international nuclear non-proliferation laws? What concrete steps can be taken to address the issue of institutional fragmentation resulting from the fuel-specific approach in the creation of international regulations, to facilitate the harmonized regulation of dual-fuel and multi-fuel ships related to nuclear energy in the future?

## Figures and Tables

**Table 1:** Technical properties of nuclear-powered merchant ships.

Ship	Lenin	NS Savannah	Otto Hahn	Mutsu	Arktika	Sibir	Rossiya	Sevmorput	Sovetskiy Soyuz	Taimyr	Vaygach	Yamal	50 Let Pobedy	Arktika	Sibir
Commission year-Decommission year	1959–1989	1962–1972	1968–1979	1974–1992	1975–2008	1977–1992	1985–2013	1988–present	1989–2014	1989–present	1990–present	1992–present	2007–present	2020–present	2021–present
Flag state	Soviet Union	United States	Germany	Japan	Russia	Russia	Russia	Russia	Russia	Russia	Russia	Russia	Russia	Russia	Russia
Type	Icebreaker	Cargo and passenger demonstration ship	Cargo and research ship	Cargo ship	Icebreaker	Icebreaker	Icebreaker	Cargo ship	Icebreaker	Icebreaker	Icebreaker	Icebreaker	Icebreaker	Icebreaker	Icebreaker
Length over all (m)	134	181.6	172	130	150	150	150	260.3	150	152	152	150	151	173	173
Beam (m)	27.6	23.8	23.4	19	30	30	30	32.2	30	29	29	30	30	34	34
Gross Tonnage	11,620	15,858	16,870	8240	20,646	20,646	20,646	38,226	20,646	20,791	20,791	20,646	23,440	33,540	33,540
Deadweight tonnage	3073	9570	14,079	2400	2750	2750	2750	26,480	2750	3550	3550	2750	3505	9000	9000
Displacement tonnage (t)	16,000	22,000	25,790	10,400	23,500	23,500	23,500	61,800	20,650	21,100	21,100	23,455	25,840	33,540	33,540
Speed (kn)	18	21	17	17.2	20.6	20.6	20.6	21	21	20	20	22	21	22	22
Engine (power)	OK-150→OK-900/A nuclear reactors (270 MW→342 MW)	“Babcock & Wilcox” pressurised water nuclear reactor (74 MW)	pressurised water nuclear reactor (38 MW)	pressurised water nuclear reactor (36 MW)	OK-900A nuclear reactors (342 MW)	OK-900A nuclear reactors (342 MW)	OK-900A nuclear reactors (342 MW)	KLT-40 nuclear reactor (135 MW)	OK-900A nuclear reactors (342 MW)	KLT-40M nuclear reactor (135 MW)	KLT-40M nuclear reactor (135 MW)	OK-900A nuclear reactors (342 MW)	OK-900A nuclear reactors (342 MW)	RITM-200 nuclear reactors (350 MW)	RITM-200 nuclear reactors (350 MW)
Propulsion power (MW)	34	16.4	8	7.5	52.8	52.8	52.8	29.4	52.8	74.8	74.8	52.8	52.8	110	110
Fuel (LEU = low-enriched uranium; HEU = highly enriched uranium)	LEU, 5% enriched→HEU, 20–90% enriched	UO2, 4% enriched	LEU, 3.5–6.6% enriched	LEU, 3.24–4.44% enriched	HEU, 90% enriched	HEU, 90% enriched	HEU, 90% enriched	HEU, either 30–40% or 90% enriched	HEU, 90% enriched	HEU, either 30–40% or 90% enriched	HEU, either 30–40% or 90% enriched	HEU, 90% enriched	HEU, 90% enriched	HEU, 20% enriched	HEU, 20% enriched

**Table 2:** International conventions concerning the regulation of nuclear propulsion related marine environmental risks.

Category	Conventions
Nuclear safety conventions	1979 Convention on the Physical Protection of Nuclear Material
	1986 Convention on Early Notification of Nuclear Accident
	1986 Convection on Assistance in the Case of a Nuclear Accident or Radiological Emergency
	1994 Convention on Nuclear Safety
	1997 Joint Convention on the Safety of Spent Fuel Management and on the Safety of Radiation Waste Management
Nuclear liability conventions	1960 Convention on Third Party Liability in the Field of Nuclear Energy (Paris Convention)
	1963 Vienna Convention on Civil Liability for Nuclear Damage (Vienna Convention)
	1963 Convention Supplementary to the Paris Convention (Brussels Supplementary Convention)
	1997 Convention on Supplementary Compensation for Nuclear Damage (CSC)
Maritime transport safety and liability conventions	1962 Convention on the Liability of Operators of Nuclear Ships (Brussels Nuclear Ship Convention)
	1965 International Maritime Dangerous Goods Code (IMDG Code)
	1971 Convention Relating to Civil Liability in the Field of Maritime Carriage of Nuclear Material
	1974 International Convention for the Safety of Life at Sea (SOLAS) 1978 International Convention on Standards of Training, Certification and Watchkeeping for Seafarers (STCW)
	1981 Code of Safety for Nuclear Merchant Ships 2015 International Code of Safety for Ships Using Gases or Other Low-Flashpoint Fuels (IGF Code)
Radioactive marine pollution control related conventions	1972 Convention on the Prevention of Marine Pollution by Dumping of Wastes and Other Matter (London Convention)
	1973 International Convention for the Prevention of Pollution from Ships (MARPOL)
	1982 United Nations Convention on the Law of the Sea (UNCLOS) 2000 Protocol on Preparedness, Response and Co-operation to Pollution Incidents by Hazardous and Noxious Substances (OPRC-HNS Protocol)

## Data Availability

No new data were created or analyzed in this study. Data sharing is not applicable to this article.
